# Photoluminescence Emitter with 100% Power Efficiency—The Key Role of the Absorption Edge in CsPbBr_3_ Perovskite Quantum Dots

**DOI:** 10.1021/acsnano.6c02074

**Published:** 2026-05-12

**Authors:** Jan Valenta, Michael Greben, Toranosuke Takagi, Martin Vacha

**Affiliations:** † Charles University, Faculty of Mathematics and Physics, Department of Chemical Physics and Optics, Ke Karlovu 3, CZ-12000 Prague 2, Czechia; ‡ School of Materials and Chemical Technology, Institute of Science Tokyo, 2-12-1 Ookayama, Meguro, Tokyo 152-8552, Japan

**Keywords:** perovskite nanocrystals, luminescence, radiometry, anti-Stokes, radiative cooling, absorption edge

## Abstract

Photoluminescence (PL) power efficiency, represented by the ratio of emitted to absorbed light energy, is a crucial factor for applications like radiative cooling. Yet, unlike PL quantum yield, achieving near-100% power efficiency in PL emitters remains mostly elusive. Here, we use spectrally resolved absolute radiometry method to study the PL quantum yield and power efficiency of solution-dispersed CsPbBr_3_ quantum dots (QDs). The samples were optimized by ligand engineering and controlled aging over the span of several months. Absorption edge changes reveal that the aging causes self-healing of intraband defect states that are otherwise contributing to a decrease of the PL quantum yield. In the optimized samples, we observed PL quantum yield reaching 100% and the PL power efficiency also approaching unity. This result means that all the absorbed excitation light energy is reemitted as luminescence. For the excitation wavelength of 532 nm, the emitted light energy comprises ∼ 80% of anti-Stokes PL and 20% of Stokes-shifted PL, while for the wavelength of 543 nm, the emission is composed entirely of anti-Stokes PL. These parameters are promising for many potential advanced applications, such as radiative cooling.

## Graphical abstract

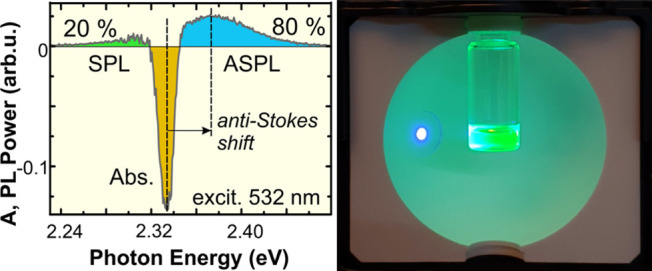

Photoluminescence (PL) is a radiative relaxation of electronic excited states in solutions and condensed matter.^1^ This phenomenon forms the basis of many important applications in lighting,^2^ signaling, light harvesting,^3,4^ super-resolution microscopy,^5^ bioimaging, etc. Typically, there is a competition between radiative and nonradiative processes, which leads to dissipation of some of the absorbed power into heat. An inherent power loss arises from the energy difference between absorbed and emitted photons—the so-called Stokes shift—which results from energy relaxations within the excited states.^1^

The development of better luminophores continues. One approach to reduce the nonradiative relaxation is the fabrication of semiconductor nanoparticles, known as quantum dots (QDs), in which the transition oscillator strength can be enhanced by the quantum confinement effect and the nonradiative centers can be largely eliminated.^6^ Some QD materials, like CdS or CdSe with an optimized core/shell structure, are approaching a 100% PL quantum efficiency^7^ (PLQY, defined as the ratio of the number of emitted to absorbed photons). More recently, metal-halide perovskite (MHP) QDs have been shown to exhibit excellent luminescent properties.^8^ Perovskites feature good carrier mobility, high defect tolerance, and very strong electron–phonon interaction, although they are prone to photochemical degradation.^9^ Owing to extensive research efforts, applications of MHP in LEDs,^10^ solar cells,^11,12^ scintillators,^13^ etc., appear highly promising.

A particularly specific application of luminescent materials is radiative cooling (RC). One can distinguish between relative and absolute radiative cooling. Relative (or passive) RC refers to the reduction of surface heating by absorbed light through efficient PL emission. As a result, the temperature of the surface is lower than it would be without the cooling applied. Recently, there has been an increasing interest in applying daytime luminescent materials for the RC of urban surfaces as a strategy to mitigate the urban heat island effect.^14,15^ The absolute RC originates from an old idea^16^ and refers to actively lowering the temperature of an object below the ambient temperature. It is only possible when the emitted photon has higher energy than the absorbed one—i.e., only when the Stokes shift is reversed. This situation can occur in multiphoton processes, which are, however, usually also accompanied by energy relaxation.^17^ In principle, the excitation of PL at the absorption edge can lead to the emission of photons with higher energy—anti-Stokes (AS) PL. Such ASPL photons harvest the energy of phonons (vibrations), and there has been continued interest in realizing RC in condensed phases.^18^ A successful RC system was demonstrated by utilizing thermal energy absorption within a Stark manifold of Yb^3+^ ions doped into glass^19^ or crystal matrices.^20^ However, even though the rare earth-based systems succeed in cooling down to cryogenic temperatures, they face a problem of minimal achievable temperature (of about 90 K for Yb/YLF).^18^ Recent attention has therefore turned to semiconductors, and intriguing reports on successful RC using CdS nanobelts^21^ or MAPbI_3_ (3D) and PhEPbI_4_ (2D) lead-halide perovskites^22^ have appeared; the debate over these results is ongoing.^23,24^ Still, MHP QDs appear as a most promising material for RC, as highly efficient ASPL has been observed independently by different groups.^25–27^

In this work, we study the absolute PLQY and power efficiency (PLPE, defined as the ratio of emitted to absorbed light energy) of CsPbBr_3_ QDs in liquid suspension by directly measuring the absorbed and emitted light energies using absolute optical radiometry with an integrating sphere. Maximized PLPE (in addition to near-unity PLQY) is a necessary precondition for efficient absolute radiative cooling. We observed PLQY reaching 100% and the total PLPE approaching 100% as well. With the excitation at 532 nm (2.33 eV), the PLPE comprises roughly 80% of ASPL and 20% of Stokes-shifted PL (SPL), while for 543 nm (2.28 eV) excitation, the near-unity PLPE is 100% due to ASPL (with a larger uncertainty). These extraordinary values are achieved by QD surface engineering using DDAB ligands and controlled sample aging that causes defect healing, as revealed by changes in the absorption edge.

## Results and Discussion

### Optical Characterization—Linear Behavior of ASPL

The CsPbBr_3_ samples studied were synthesized in two batches under identical conditions, each producing QDs with oleic acid (OA)/oleylamine (OLA) passivation (samples OA(1) and OA(2)). Parts of each batch were then ligand-exchanged to didodecyldimethylammonium bromide (DDAB) for better surface passivation (samples DDAB(1) and DDAB(2)). The successful ligand exchange has been confirmed by FTIR measurements,^28^ which clearly show disappearance of the vibrational peaks corresponding to oleic acid and oleylamine, respectively, in the DDAB samples (Figure S1). TEM characterization of samples synthesized under the same conditions and ligand-exchanged with DDAB is presented in Figure S2.

All four samples reveal similar optical properties (Figure 1a), with a characteristic absorption spectrum and strong green PL band with a peak at 522 nm, which is Stokes shifted from the excitonic absorption peak by about 60 meV. When the excitation wavelength is tuned within the PL band, part of the PL appears at shorter wavelengths (higher photon energies)—this is ASPL, whose spectral shape and position are almost identical to those of the SPL band. The stability of the PL peak position (∼2.375 eV) with respect to the excitation tuned from 2.25 to 2.54 eV is illustrated in Figure 1b (the horizontal line). In this figure, the absorption and PL peak positions refer to apparent positions as detected in the integrating sphere, as illustrated in the inset in Figure 6b. The absorption peak (energy absorbed by the sample, orange symbols) shifts linearly with the photon energy of the excitation source as it is tuned within the absorption band. When the excitation peak is located within the range of the PL band, the emission band splits into the separate ASPL (blue symbols) and SPL (green symbols) bands. The apparent positions of these bands change as the laser is tuned because part of the emission around the excitation peak is missing due to the absorption. Importantly, outside the range of the PL band, both the ASPL and SPL peaks show the same energy that is constant.

**Figure 1:**
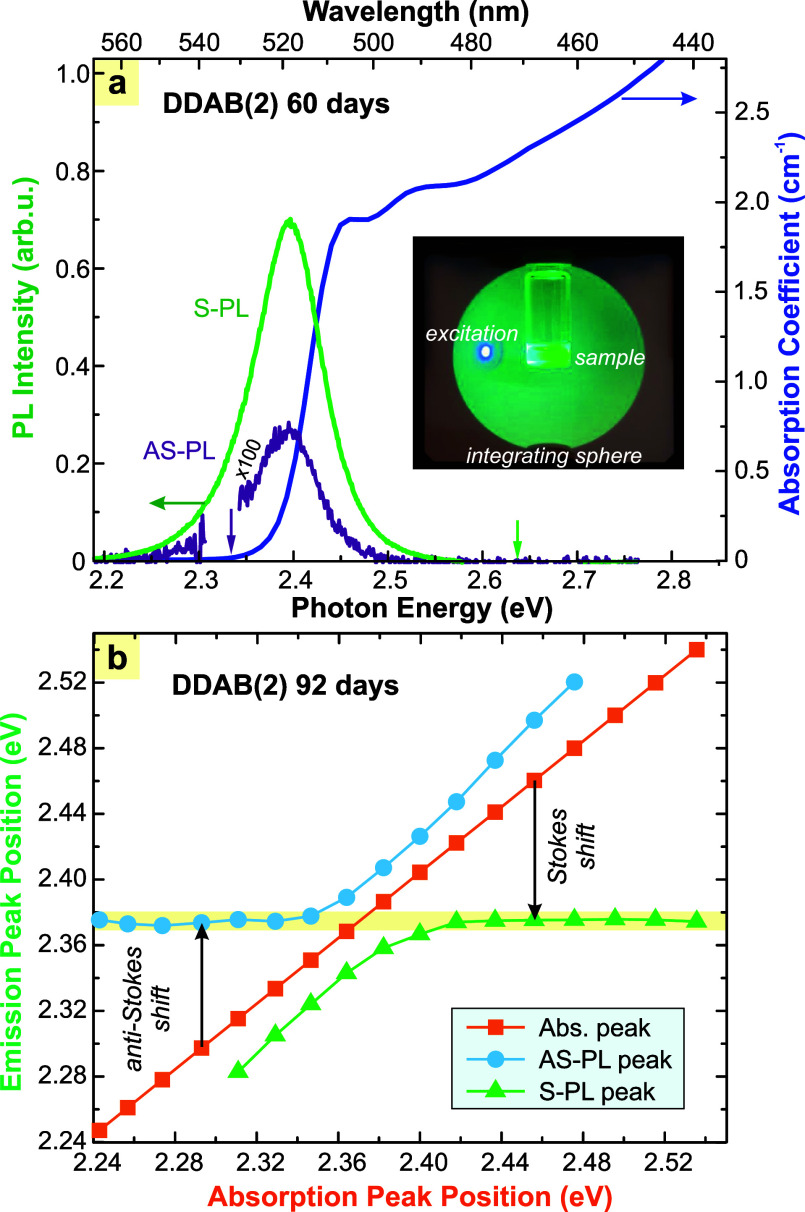
(a) Absorption (blue) and luminescence (green—excited 405 nm, violet—excited 532 nm) spectra of DDAB(2) QDs. The inset presents a photo of the open integrating sphere with a sample in the middle—this is the experimental configuration of PL measurements (with the closed IS, obviously). (b) The PL peak position as a function of absorption (excitation) photon energy. The yellow horizontal stripe highlights the stability of the PL peak at ∼2.375 eV.

In the radiometry, we use continuous wave (cw) excitation with low-to-moderate excitation power. In the case of laser diodes (405 or 532 nm), the maximum excitation power density reaches a few mW/cm^2^ (the direct excitation in the center of an integrating sphere); for the LDLS (laser-driven light source) tunable excitation, the power density is 3 orders of magnitude lower. Both SPL and ASPL intensities have linear power dependence on excitation power (see Figure 2a for ASPL) within the accessible range up to ∼5 mW/cm^2^. This indicates the absence of any nonlinear processes like multiphoton excitation or multiexcitonic effects. Actually, we can estimate the average number of excitons ⟨*N*⟩ per QD as a product of photon flux Φ_p_, absorption cross section σ, and average PL decay time τ. Let us take the decay time of 10 ns (Figure 2b) and the absorption cross section in the range of 10^–13^–10^–14^ cm^2^ (according to refs 29,30 ). Then, for the typical tunable excitation by LDLS of 4 μW/cm^2^, we obtain very low occupation ⟨*N*⟩ = 1 × 10^–8^–1 × 10^–9^, as expected. Even for the strongest excitation of ∼5 mW/cm^2^ (Figure 2a), we get the estimated exciton population per QD orders of magnitude below 1 (10^–5^–10^–6^).

**Figure 2:**
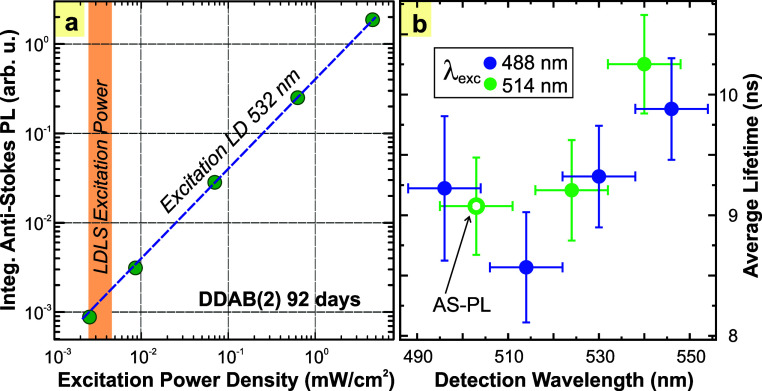
(a) The power dependence of PL intensity (excited with a laser diode at 532 nm) reveals linear dependence. (b) The average PL decay time excited at either 488 or 514 nm. ASPL has the same decay time as SPL at the same emission wavelength (indicated by the arrow).

The PL decay kinetics slightly change depending on the emission wavelength (the average decay time is between 8.5 and 10.2 ns). The average decay times were obtained from fitting the decays with three exponentials (see Figure S5). On the single-QD level, the decay is single-exponential,^31^ and the unity PLQY should, in principle, result in the single-exponential decay in a homogeneous sample. However, as shown in Figure S2, there is a distribution in the sizes of the measured QDs and therefore also distributions of the radiative rates and lifetimes that give rise to the multiexponential decay. ASPL has the same decay time as SPL at the same emission wavelength (around 500 nm) (see Figure 2b). This fact indicates that both SPL and ASPL follow the same recombination paths. The slight dependence of the decay kinetics on the detection wavelength is often observed also in other QD systems (see, e.g., our recent work on InP/ZnS for a detailed description^32^). We suppose this is due to the non-negligible size distribution of QDs (Figure S2) and the related variation of the weak quantum confinement effect.

### Aging of the CsPbBr_3_ QD Suspensions

To assess the effect of aging on defect healing in the CsPbBr_3_ QDs, we repeated the PLQY and PLPE characterization on all samples for about 6 months with an interval of several weeks. The stock samples were stored in a refrigerator at 7 °C in dark, and for each measurement, we took a “virgin” sample from the stock of the same concentration.

The general evolution of the PLQY (Figure 3) is similar for all four samples. During the first two months, the values of PLQY increase, reaching 100% for the DDAB-passivated samples and keeping at this level for two more months. Then, from the fourth month onward, a slow decrease in PLQY is observed.

**Figure 3:**
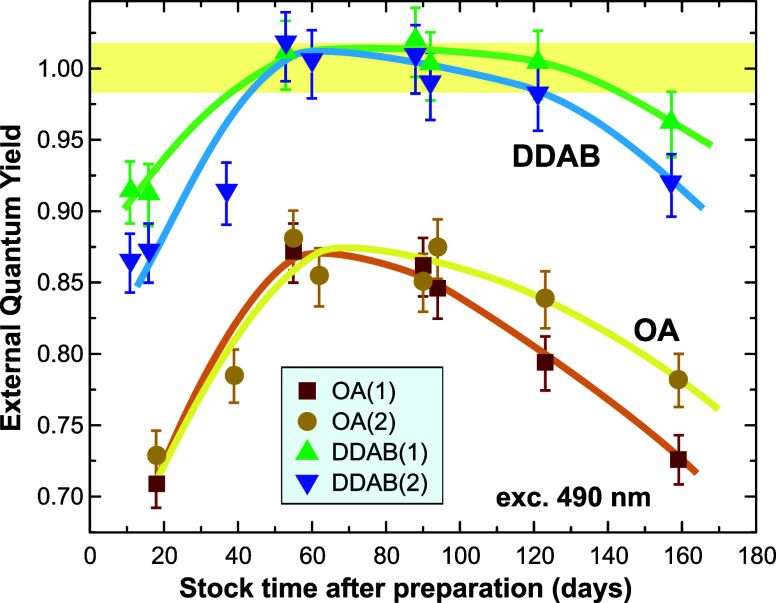
Long-term evolution of PLQY of both sample batches and passivations (excited at 490 nm).

Photophysical changes of OA samples have been recently studied on the level of single QDs.^33^ With aging, the samples converge toward a narrower size distribution and reduced spectral and blinking heterogeneity. Since the healing trend between the OA and DDAB samples is very similar, we assume in analogy with ref [28] that in the as-synthesized samples, there are numerous QD aggregates where self-reabsorption and energy and/or charge transfer to the lowest band gap QD reduce the PLQY. Over time, the extra ligands in the solution contribute to slow dispersion of the aggregates and continued surface passivation, which are complete in about 60 days. The following PLQY decrease is most probably caused by defects created by oxygen dissolved in toluene.

During the aging, remarkable changes occur at the absorption edge (Figure 4a). In perovskites, the absorption edge is known to exhibit an exponential shape, the so-called Urbach tail (UT),^34^ which reflects strong electron–phonon interaction. The UT is described by the following equation^35^

1 $$\alpha \left(E\right)=\alpha_{0}\exp\left(\frac{E-E_{0}}{E_{U}}\right)$$

where *E*_0_ and *E*_U_ are the Urbach focus energy and the Urbach energy, respectively, and α_0_ is a constant. By fitting the absorption edge of the DDAB(2) sample measured 92 days after preparation, we obtain *E*_0_ = 2.257 ± 0.008 eV and *E*_U_ = 18.4 ± 0.4 meV (see the dashed orange line in Figure 4a). A more detailed discussion of the UT fitting values is provided later.

**Figure 4:**
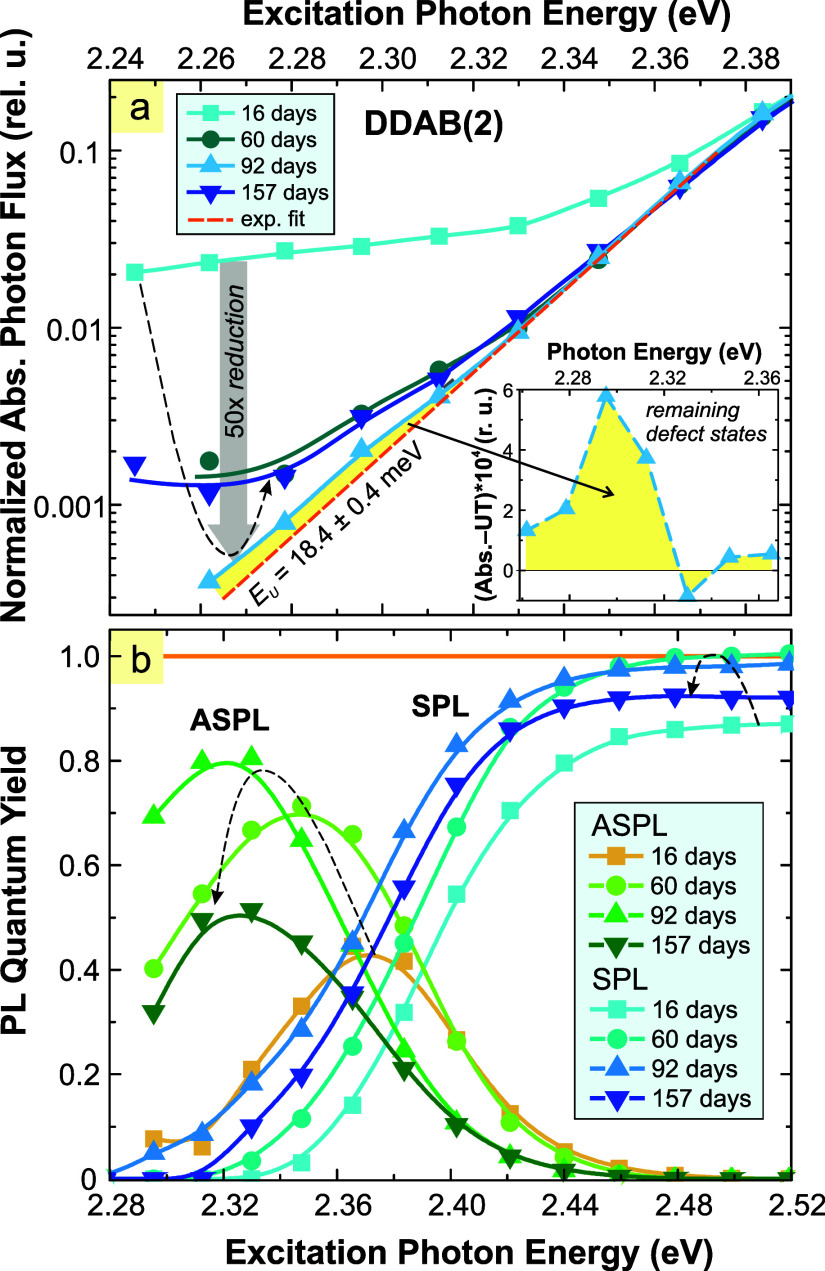
(a) The evolution of the absorption photon flux (normalized at 470 nm, 2.64 eV). The extended absorption tail decreases, reaching a minimum 3 months after preparation and then starts to increase again. The orange dashed line is the Urbach edge fit, and the yellow area is the absorption residuum of defect states (separately shown in the inset). (b) The corresponding evolution of SPL and ASPL QY with time. The maximum PLQY is reached after 3 months.

An additional absorption tail is often observed in perovskites of various compositions and forms, from bulk to QDs. This additional tail is less steep, is superimposed on, and can extend far from the UT. The presence of this band indicates states related to defects within the band gap and resembles the tail states in disordered semiconductors.^36^ We observe such a tail in fresh samples (Figure 4a), where it reaches an amplitude of about 1/50 of the absorption maximum at 470 nm. Its amplitude decreases with time and becomes almost negligible in the optimal (3–4 months old) sample, with a small residuum as seen from the yellow area in Figure 4a and the inset. After this period, the additional absorption starts growing slowly again.

The aging of the samples is also manifested by the PLQY changes (Figure 4b). The SPL QY increases up to 100% for excitation at photon energies above 2.48 eV in the optimally aged sample (2–4 months). PLQY of ASPL is increasing and red shifting with aging due to reduced reabsorption and improving yield, reaching the peak PLQY of around 80% for a photon energy of 2.32 eV. After four months, both SPL and ASPL QY start to decrease in concordance with the absorption changes.

To confirm that the PLQY variations observed over several months are not due to changing concentrations in the nanocrystal sample solutions, we measured absorption spectra during the aging and found no changes over the first 3 months. The absorbance dropped by about 15% after 5 months, as a result of the formation of defects and overall degradation (see Figure S3).

### Detailed Properties of ASPL

An overview of PL spectra under excitation tuned from 488 to 536 nm (2.54–2.31 eV) is presented in Figure 5 in both linear (a) and logarithmic (b) scales. The PL band shape is remarkably stable regardless of the excitation wavelength. The inset in Figure 5a shows that a clearly detectable PL band is present for excitation up to 552 nm, i.e., for the shift between excitation photon energy and the ASPL peak of about 125 meV.

**Figure 5:**
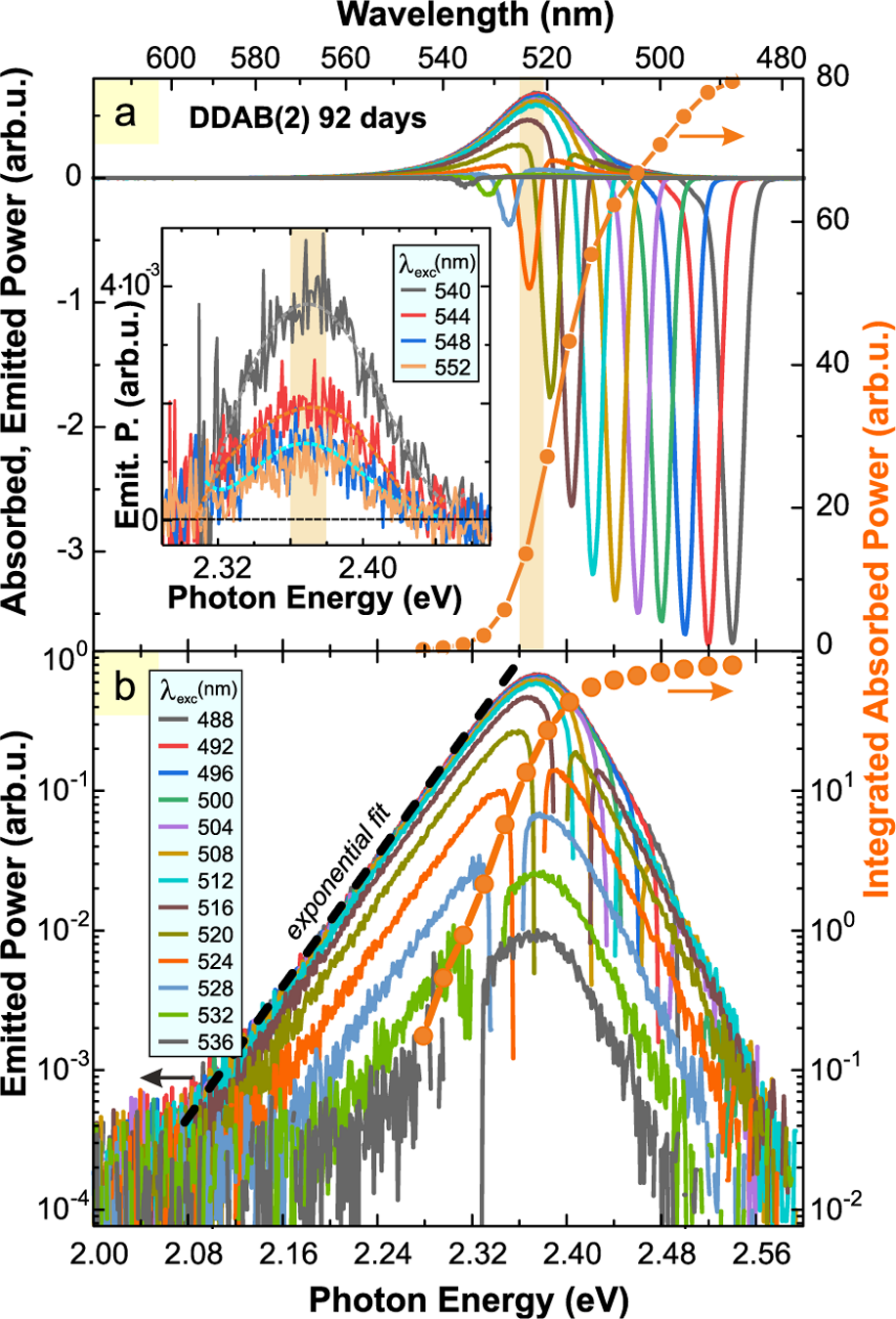
(a) The emission/absorption spectra used for calculation of PL power efficiency under excitation tuned from 488 to 536 nm (step 4 nm). The negative and positive parts correspond to the absorbed and emitted powers, respectively. The inset shows the ASPL band still visible under excitation at 510–552 nm. The orange line with dots is the absorption edge. (b) The same spectra as in panel (a) are plotted in a logarithmic intensity scale. The PL edge for the 488 nm excitation is fitted by the exponential function described by eq 2.

The corresponding absorption edge (taken from Figure 4a) is plotted over the PL spectra (orange line with dots) in order to illustrate its overlap with the PL band. The absorption tail, which is formed by the extended absorption states, plays two contradictory roles: (i) it allows for the excitation of ASPL and, at the same time, (ii) it reabsorbs the emitted PL photons. In fact, the sequence of reabsorption and reemission events continues until the excitation is lost through nonradiative recombination or a photon leaves the sample. This process is called photon recycling.^37,38^ The PLQY must be very high in order for the photons to have a good chance of surviving the recycling. The defect-related absorption tail thus evidently reduces the PLQY (Figure 4b).

The PL spectral band has clear exponential tails on both sides (Figure 5b). The low-energy PL tail shape *I*_PL_ can be described by the following equation^39^

2 $$I_{PL}\left(E,T\right)≅E^{2}\exp\left[E\left(\frac{1}{E_{U}}-\frac{1}{k_{B}T}\right)\right]$$

The slope of the exponential edge depends not only on the Urbach energy *E*_U_ but also on the thermal energy *k*_B_ *T*. This temperature dependence of the PL edge was utilized to reveal temperature changes in the study of the MHP QD radiative cooling.^40^ In our case, we expect no excitation-induced heating to take place as we used very low excitation power (a few μW; Figure S6). The sample temperature is assumed to be equal to the ambient laboratory temperature (19 °C, 292 K), and the thermal energy is thus 25.16 meV. Using this thermal energy, the fit of the PL edge (Figure 5b) yields the Urbach energy of 15.2 ± 0.5 meV. This value is close to that reported for CsPbBr_3_ QDs in the literature,^41,42^ but it is significantly lower than the 18.4 meV obtained above by fitting the absorption edge (Figure 4a). Similar observation was already described by Lytle et al. with *E*_U_ value (from a PL edge) in excellent agreement with our value.^42^

We have to stress that measurement of absorbance in an integrating sphere does not provide the absorption coefficient (used to describe the Urbach tail in eq 1) because the light pass distance through a sample is not precisely known and it depends on the wavelength. The determined absorbed photon flux still follows the exponential profile for low absorbance values, but the fitted slope may deviate from the correct Urbach energy. In addition, the effect of photon recycling is also enhanced by reflections within the integrated sphere (see Figure S8). These disadvantages are outweighed by the great advantage of directly obtaining both emitted and absorbed photon flux in terms of power, enabling the calculation of the PLQY and PLPE.

In Figure 6a, we plot the integrated power of the data from Figure 5 as a function of the excitation photon energy. The resulting power efficiencies for SPL, ASPL, and total PL are shown in Figure 6b. The shift of the excitation to longer wavelengths gradually transforms SPL to ASPL because the spectral position of the PL band is very stable. One can see that for the excitation at 2.33 eV (532 nm), the power efficiency is 100%. For even lower photon energies of 2.28 eV (543 nm), the efficiency is also close to unity; however, the uncertainty (determined statistically from repeated measurements) increases as the absorption becomes very low. For comparison of PLQY in OA- and DDAB-passivated samples, see Figure S9.

**Figure 6:**
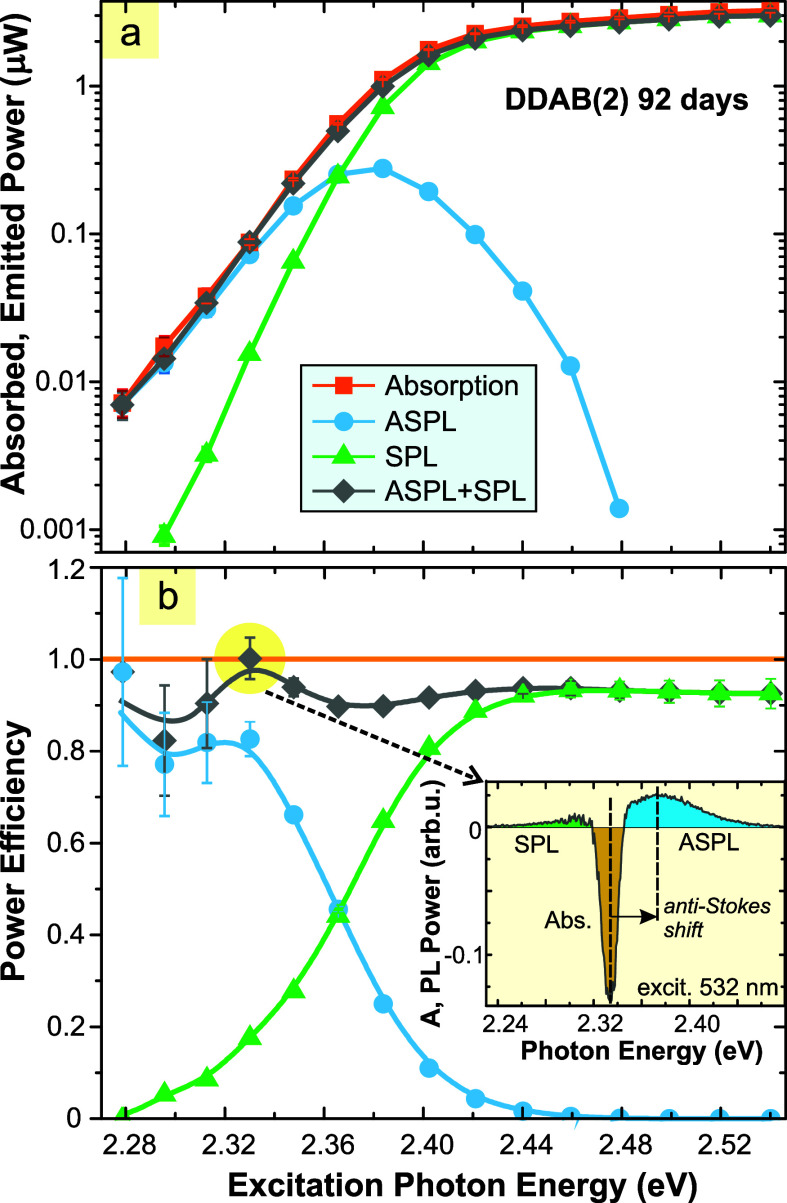
(a) Absorbed (orange) and emitted (blue—ASPL, green—SPL, and gray—AS + SPL) power as a function of excitation photon energy. (b) Power efficiency for both PL components and total PL (gray line with squares). The unity efficiency at 2.33 eV (532 nm) is highlighted by a yellow circle. The inset shows the corresponding power spectra.

Let us note that the general evolution of PLQY with excitation wavelength is sometimes described by the so-called Kasha–Vavilov rule. This rule states that the quantum yield is constant—independent of the excitation wavelength (photon energy).^43^ In the case of strong ASPL, we have to consider the total yield (or efficiency) SPL + ASPL. From Figure 6b, we can see that the Kasha–Vavilov rule still holds when ASPL contributes a major part of the total emission.

## Conclusions

Using the absolutely calibrated radiometry setup with an integrating sphere, we followed the evolution of absorption and PL characteristics of CsPbBr_3_ QDs over time. In DDAB passivated samples with optimal aging (2–4 months), we observed SPL quantum yield reaching 100% for excitation photon energy >2.44 eV. The ASPL reaches the peak PLQY of 80% for excitation at 2.33 eV (532 nm), and the total (AS + S) PL power efficiency at this excitation reaches unity. This remarkable result means that all the absorbed excitation light energy at 532 nm is reemitted as luminescence (comprising roughly 80% of ASPL and 20% of SPL; Figure 6b). For still longer wavelengths, where the emission is composed entirely of ASPL (532–543 nm), the PLPE stays near-unity, but the experimental uncertainty becomes large due to very low absorbance.

In addition, we are currently carrying out a single-particle study of the CsPbBr_3_ QDs and succeeded in ASPL detection from individual QDs of the DDAB sample. An example of the data is shown in Figure S4. Complex single-particle characterization in the future will further strengthen the discussion of the ASPL-related photophysics.

The present experiments also allowed us to reveal the changes in the absorption edge. The fresh samples have an extended absorption tail, in addition to the normal Urbach tail. This defect-related tail almost disappears within two months after preparation and starts to grow again after four months. Correlation of these changes with the trends in PLQY and PLPE points to the origin of the decreased PLQY and PLPE as due to intra-band-gap defect states. The Urbach tail states not only enable the excitation of anti-Stokes PL but also cause photon recycling (reabsorption). The perfect PLQY is thus necessary for the PL photons to maximize their chance to survive recycling and leave the sample. The PLQY is suppressed by the additional defect-related tail states (intraband defects) that increase reabsorption.

We observed no signs of radiative cooling, such as changes of the PL band edge with increasing excitation power.^39^ Our samples have the form of large ensembles of QDs in toluene—the thermal capacity of such large reservoir does not allow for a change in temperature with very low radiative power involved.

The radiometry setup used in this study does not allow for varying the temperature of the sample. We plan to build a setup enabling PLQY measurement at variable temperatures in the future. Still, the photophysical parameters of the CsPbBr_3_ QDs optimized by ligand engineering and aging and revealed by absolute radiometry are very promising for possible radiative-cooling applications.

## Methods

### Preparation of CsPbBr_3_ QDs

#### Synthesis of CsPbBr_3_

CsPbBr_3_ QDs were synthesized as reported previously^8^ with slight modifications. Details are described in the Supporting Information. Briefly, the Cs precursor was synthesized using Cs_2_CO_3_, oleic acid, and 1-octadecene (ODE) under a N_2_ atmosphere upon heating at 150 °C. The PbBr_2_ precursor was prepared by mixing PbBr_2_ with ODE, oleic acid, and oleylamine at 140 °C under a N_2_ atmosphere. The Cs precursor solution was injected in the PbBr_2_ precursor at 140 °C, and the obtained yellow-green solution was quenched in an ice bath. The synthesized CsPbBr_3_ solution was mixed with ODE and centrifuged at 12,000 rpm for 10 min. The precipitate was redispersed in the same volume of toluene and further centrifuged at 12,000 rpm for 10 min. The supernatant at the final concentration of 0.40 μM was used either as OA(1) or OA(2) samples, or as the starting compound for the ligand exchange.

#### Ligand Exchange

Details are described in the Supporting Information. Briefly, the ligand solution was prepared by mixing didodecyldimethylammonium bromide (DDAB) and PbBr_2_ in toluene and stirring at 50 °C in air. The ligand exchange reaction was carried out by adding the ligand solution to the as-synthesized CsPbBr_3_ dispersion in a volume ratio of 1:0.3 and by stirring this solution for 1 h at room temperature in air. Ethyl acetate was then added, and the solution was centrifuged at 14,000 rpm for 10 min. After redispersing the precipitate in toluene, ethyl acetate was again added and centrifuged under the same conditions. The precipitate was finally redispersed in toluene at a final concentration of 0.44 μM and used as the DDAB(1) or DDAB(2) samples.

### Optical Characterization of CsPbBr_3_ QDs

The transmission spectra of suspensions were measured using a double-beam spectrometer (Specord 250, Analytik Jena).

Time-resolved (TR) PL decay kinetics was acquired using the optical microscope (Leica TCS SP8) with the time-correlated single photon counting (TC-SPC) detection and sub-nanosecond excitation pulses. A low-magnification objective lens (10×, NA = 0.3) was used to deliver excitation and collect PL of a sample in the glass vial. The PL signal was detected using three Leica SP8 HyD detectors. The instrument response function (IRF) was in sub-nanosecond scale, which was significantly faster than PL decay kinetics and therefore its deconvolution was unnecessary. TR PL data were fitted by a three-exponential function (representing the lifetime distribution in the QD ensemble; Figure S5) in order to calculate the average lifetime (Figure 2b).

#### Optical Radiometry

The absolute PLQY and PLPE were determined using a setup based on an integrating sphere (IS) with a diameter of 50 mm (Thorlabs). The samples (with a typical volume of 0.3 mL) are placed in disposable glass vials and mounted on a holder in the center of IS. The tunable excitation source is based on the laser-driven light-source (LDLS, Energetiq) coupled to a 15 cm monochromator (Acton SP-2150i). The detection part consists of the 30 cm imaging spectrograph (Acton SP-2300i) with the LN-cooled back-illuminated CCD camera (Spec-10:400B, Princeton Instruments). Both the excitation and the emission signals are coupled and guided using the silica fiber bundles. The tunable source excitation is weak ∼4 μW/cm^2^ at the place of a sample in IS (Figure S6). For stronger excitation, we used small diode laser modules 405 or 532 nm (Thorlabs). The spectral response of the setup was calibrated using the standard of spectral irradiance (a tungsten-halogen lamp 45 W, Newport Oriel). The resulting sensitivity curve *C* (λ) relates the measured signal (in counts per second per pixel) to the input spectral light power (in W/nm). All measurements presented here were performed at room temperature (∼19 °C).

Calculation of PLQY or PLPE is based on repeated measurements of a tested (S) and reference (R) samples for each excitation wavelength. The reference sample is the same type of glass vial with an equivalent volume of solvent (toluene). The PL efficiency η, PLQY or PLPE, is then calculated using the following equations^44^

3 $$\eta_{QY}=\frac{\int_{em.band}\frac{I_{S}\left(\lambda_{em}\right)-I_{R}\left(\lambda_{em}\right)}{C\left(\lambda_{em}\right)\cdot hc/\lambda_{em}}d\lambda_{em}}{-\int_{ex.band}\frac{I_{S}\left(\lambda_{ex}\right)-I_{R}\left(\lambda_{ex}\right)}{C\left(\lambda_{ex}\right)\cdot hc/\lambda_{ex}}d\lambda_{ex}}$$

4 $$\eta_{PE}=\frac{\int_{em.band}\frac{I_{S}\left(\lambda_{em}\right)-I_{R}\left(\lambda_{em}\right)}{C\left(\lambda_{em}\right)}d\lambda_{em}}{-\int_{ex.band}\frac{I_{S}\left(\lambda_{ex}\right)-I_{R}\left(\lambda_{ex}\right)}{C\left(\lambda_{ex}\right)}d\lambda_{ex}}$$

where *I*_S_(λ) and *I*_R_(λ) are signals measured with sample and reference, respectively (the parentheses just stress the dependence on wavelength). The subscripts “em” and “ex” denote whether the spectral range relates to the emission or excitation portion of the spectrum.

The uncertainty of PLQY and PLPE is determined statistically through a sequence of repeated measurements (standard deviation). Due to the high stability of the LDLS source, the standard deviation remains about 1–2% across most of the excitation range. However, the uncertainty grows rapidly when approaching low absorption values at the absorption edge (see Figure 6b).

Further, to cross-validate the obtained PLQY values, we measured the PLQY of a reference sample of the luminophore LuAG/Ce with a nominal PLQY of 96%. The obtained PLQY value of 95.5% is in excellent agreement with the reference, providing a required cross-check. The experimental data are presented in SI as Figure S7.

## Supplementary Material



## Notes

Preparation of CsPbBr_3_ QDs: synthesis and ligand exchange; sample characterization: FTIR spectra before and after ligand exchange, TEM characterization of the DDAB sample, absorption spectra changes in time, and single-particle ASPL; time-resolved photoluminescence decay measurements: example of the PL decay curve and the fitting; optical radiometry setup testing: excitation power vs wavelength, PLQY of the standard sample LuAG/Ce; effects of photon recycling: PL spectra for the stock concentration and diluted samples inside and outside of IS; comparing PLQY of OA and DDAB passivated and optimally aged samples: comparison of PLQY for samples passivated by OA or DDAB (PDF)
