# Supplementary material for: Photoluminescence Emitter with 100% Power Efficiency—The Key Role of the Absorption Edge in CsPbBr3 Perovskite Quantum Dots
Source: ACS Nano. 2026 May 12;20(20):14709–16. doi: 10.1021/acsnano.6c02074 (PMC13218043; doi:10.1021/acsnano.6c02074)
Supplement: Supplementary file 1 [file nn6c02074_si_001.pdf]

Supporting Information

Photoluminescence emitter with 100% power efficiency – the key role of the absorption edge in $CsPbBr_3$ perovskite quantum dots

Jan Valenta[a*], Michael Greben[a], Toranosuke Takagi[b], Martin Vacha[b]

[a] *Charles University, Faculty of Mathematics and Physics, Department of Chemical Physics and Optics, Ke Karlovu 3, CZ-12000 Prague 2, Czechia.*

[b] *School of Materials and Chemical Technology, Institute of Science Tokyo, 2-12-1 Ookayama, Meguro, Tokyo, 152-8552 Japan.*

Contents:

1. ***Preparation of $CsPbBr_3$ QDs***

Synthesis of $CsPbBr_3$.

Ligand exchange

2. ***Sample characterization***

Figure S1. FTIR spectra before and after ligand exchange

Figure S2. TEM characterization of the DDAB sample

Figure S3. Absorption spectra changes in time

Figure S4. Single particle ASPL

3. ***Time-resolved photoluminescence decay measurements***

Figure S5. Example of the PL decay curve and the fitting

4. ***Optical radiometry set-up testing***

Figure S6. Excitation power

Figure S7. PLQY of the standard sample LuAG:Ce

5. ***Effects of photon recycling***

Figure S8. PL spectra for the stock concentration and diluted samples inside and outside of IS

6. ***Comparing PLQY of OA and DDAB passivated and optimally aged samples***

Figure S9. Comparison of PLQY for samples passivated by OA or DDAB (~90 days)

References

1. ***Preparation of $CsPbBr_3$ QDs***

Synthesis of $CsPbBr_3$.

$CsPbBr_3$ QDs were synthesized as reported previously[1,2] with slight modifications. The Cs precursor was synthesized using Cs_2CO_3 (Sigma-Aldrich Japan, product no. 202126, 99.9% trace metals basis) (159.7 mg), oleic acid (Sigma-Aldrich Japan, product no. 364525, technical grade, 90%) (0.5 ml), and 1-octadecene (Tokyo Chemical Industry Co., Ltd., product no. O0008, > 90.0%) (ODE, 8 ml) under a N_2 atmosphere. Upon heating, a clear solution of Cs-oleate in ODE was obtained at 150 °C, at which point the heating was stopped. The $PbBr_2$ precursor was prepared by mixing $PbBr_2$ (Sigma-Aldrich Japan, product no. 915696, Anhydrobeads™, 99.999% trace metals basis (perovskite grade)) in 10 ml ODE (139.5 mg), 0.75 ml oleic acid, and 0.75 ml oleylamine (Sigma-Aldrich Japan, product no. O7805, technical grade, 70%) at 140 °C under N_2 atmosphere. 30 s after the $PbBr_2$ precursor reached 140°C, 0.8 ml of the Cs precursor solution was injected. After 20 s, the obtained yellow-green solution was quenched in an ice bath. The synthesized $CsPbBr_3$ solution was mixed with ODE in a volume ratio of 1:1 and centrifuged at 12,000 rpm for 10 minutes. The precipitate was re-dispersed in the same volume of toluene (Kanto Chemical Co., Inc., product no. 8331-1B, > 99.7%) and further centrifuged at 12,000 rpm for 10 minutes. The supernatant was used either as OA(1) or OA(2) samples (at the concentration of 0.40 μM), or as the starting compound for the ligand exchange.

Ligand exchange

The ligand solution was prepared by adding 92.5 mg of didodecyldimethylammonium bromide (DDAB) (Tokyo Chemical Industry Co., Ltd., product no. D1974, > 98.0%) and 36.7 mg of $PbBr_2$ to 3 ml of toluene and stirring while heating at 50°C in air. The ligand solution was diluted 10 times to a final DDAB concentration of 6.7 mM, with $PbBr_2$ at the concentration of 3.3 mM. The ligand solution was added to the as-synthesized $CsPbBr_3$ dispersion (at the concentration of 0.40 μM) in a volume ratio of 1:0.3. The ligand exchanged reaction proceeded by stirring this solution for 1 h at room temperature in air. Ethyl acetate (Sigma-Aldrich Japan, product no. 09-0680, JIS special grade, ≥ 99.5%) was then added at a volume ratio of 1:1.5, and the solution was centrifuged at 14,000 rpm for 10 min. After re-dispersing the precipitate in toluene, ethyl acetate was again added and centrifuged under the same conditions. The precipitate was finally re-dispersed in toluene to achieve the concentration of 0.44 μM (adjusted by measuring absorbance at 400 nm and using literature values[3] of absorption coefficient at this wavelength), and used as the DDAB(1) or DDAB(2) samples.

2. Sample characterization

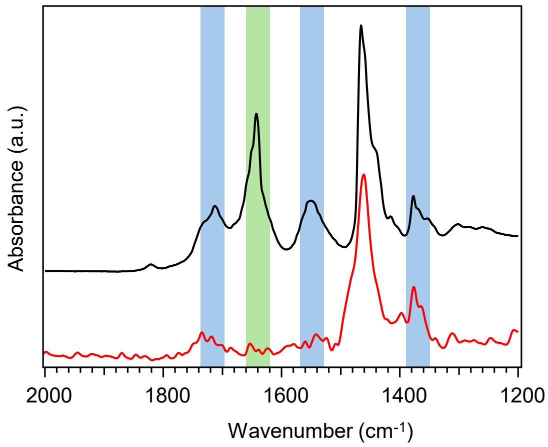

Figure S1. FTIR spectra of the OA (black line) and DDAB (red line) samples. The blue and green regions indicate frequency intervals corresponding to oleic acid and oleylamine, respectively [28].

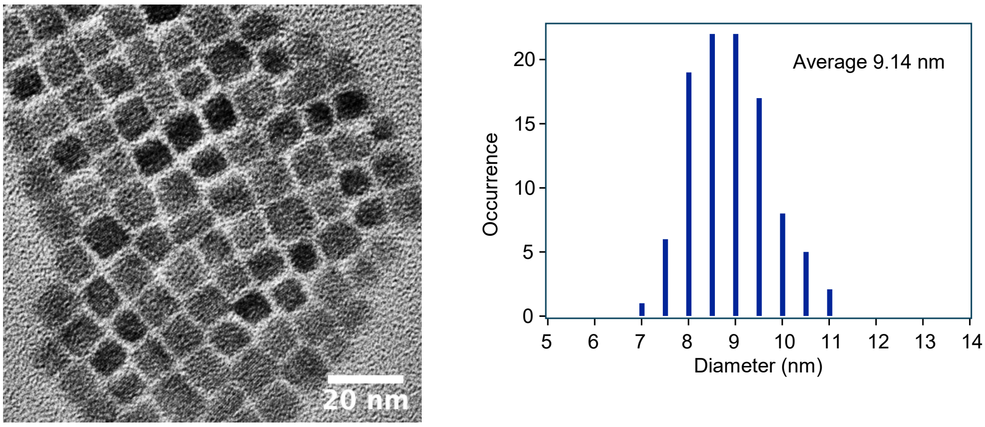

Figure S2. Left: Representative TEM image of DDAB-functionalized $CsPbBr_3$ nanocrystals. Right: Size distribution obtained by analysis of TEM images of 102 nanocrystals. The diameter of a nanocrystal is an average of two orthogonal sizes. The average of the distribution is 9.14 nm with a standard deviation of 0.86 nm.

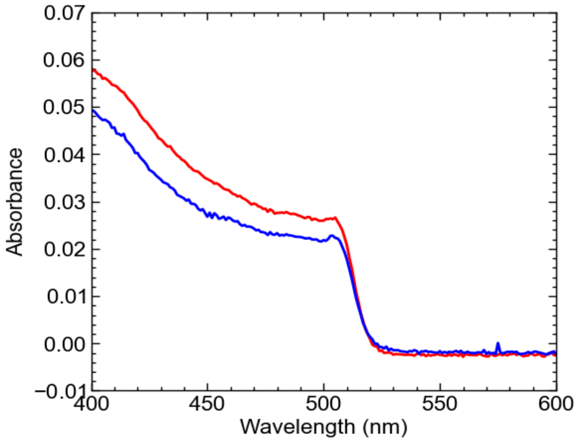

Figure S3. Absorption spectra of the DDAB samples taken after the synthesis (red line) and 5 months later (blue line)

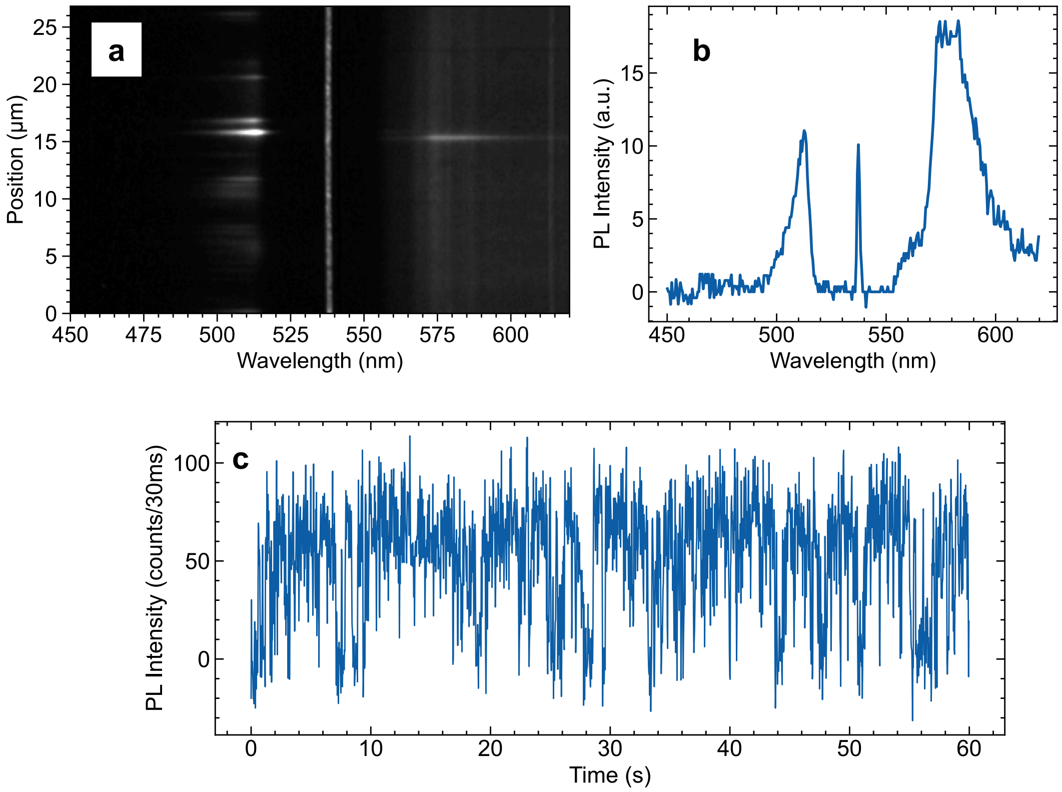

Figure S4. **a**) 2D spectral plot (position vs. wavelength) of single $CsPbBr_3$ QDs (DDAB sample) excited at 532 nm and detected using a notch filter and EMCCD camera; **b)** PL spectrum of a single $CsPbBr_3$ QD. The drop at the center is due to the notch filter blocking, the emission above 520 nm is the ASPL; **c)** Example of ASPL blinking, confirming the single particle nature of the emission.

3. Time-resolved photoluminescence decay measurements

Time-resolved (TR) PL decay kinetics was acquired using the optical microscope (Leica TCS SP8) with the time-correlated single photon counting (TC-SPC) detection and sub-ns excitation pulses. A low-magnification objective lens (10×, NA = 0.3) was used to deliver excitation and collect PL of a sample in the glass vial. The PL signal was detected using three Leica SP8 HYD detectors. The instrument response function (IRF) was in sub-ns scale which was significantly faster than PL decay kinetics and therefore, its deconvolution was unnecessary (see Fig. S5). TR PL data were fitted by a three-exponential function in order to calculate the average lifetime (Fig. 2b).

An example of the PL decay curve is shown in Fig. S5. The non-single-exponential decay curve is fitted by a three-exponential function (lifetimes τ_i and amplitudes A_i). This function should be considered as a representation of a distribution of lifetimes, which is arising from the size distribution of PQDs. The average lifetime is then calculated (Eq. s1) according to the definition of an average value of a quantity (see our review paper[4] for details on the treatment of non-single-exponential decays).

$$\bar{\tau} = \frac{\int_0^\infty tI(t)dt}{\int_0^\infty I(t)dt} = \frac{\sum_{i=1}^{N} A_i \tau_i^2}{\sum_{j=1}^{N} A_j \tau_j} \qquad \text{(s1)}$$

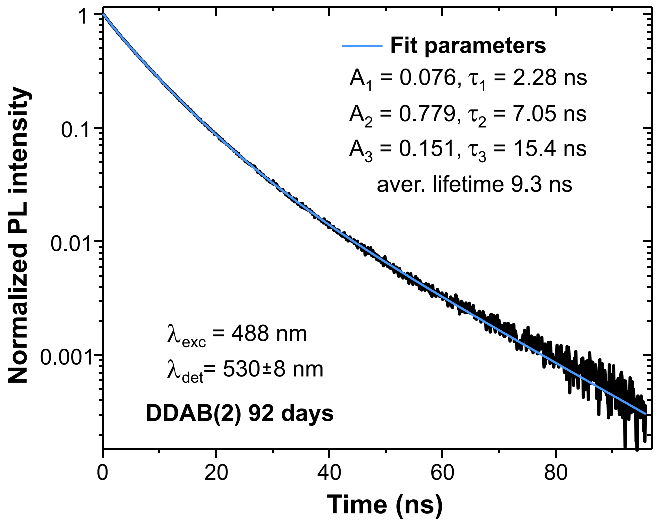

Figure S5. Example of the PL decay curve. The non-single-exponential shape of the curve is fitted by a three-exponential fit which represents the inherent lifetime distribution. Then an average lifetime is calculated by the standard method (Eq. s1).

4. Optical radiometry set-up testing

The absolute PLQY and PLPE were determined using a set-up based on an integrating sphere (IS) with a diameter of 50 mm (Thorlabs). The samples are placed in disposable glass vials and mounted on a holder in the center of IS. The tunable excitation source is based on the Laser-Driven Light-Source (LDLS, Energetiq) coupled to the 15-cm monochromator (Acton SP-2150i). The power of excitation light was measured at the output of the fiber bundle by the power-meter Coherent Fieldmaster (Fig. S6) and independently checked by the absolute calibration of the IS-set-up (using a standard of spectral irradiance).

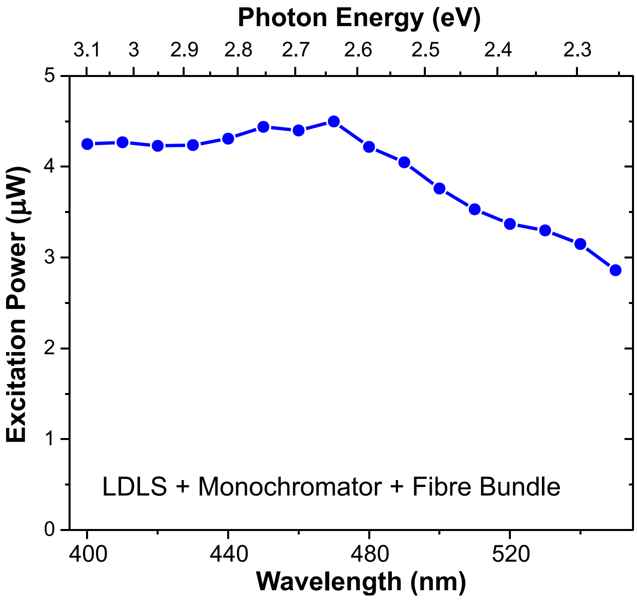

Figure S6. Excitation power (produced by the LDLS source coupled to the monochromator and the fiber bundle) entering the integrating sphere as measured by the power-meter (Coherent Fieldmaster).

The spectral response of the set-up was regularly recalibrated using the standard of spectral irradiance (45 W tungsten-halogen lamp, Newport Oriel). The correctness of the calibration and the overall methodology is cross checked by measuring an appropriate quantum yield standard. For the relevant green spectral region we use a piece of LuAG:Ce (Lutetium aluminium garnet doped with cerium, supplied by CRYTUR[5]) crystal. This material has the peak PLQY value of 0.96 (verified by an independent laboratory – Institute of Physics, CAS, Prague). Figure S7 shows PLQY for different excitation wavelengths revealing the peak value of 95.5 % at 430 nm.

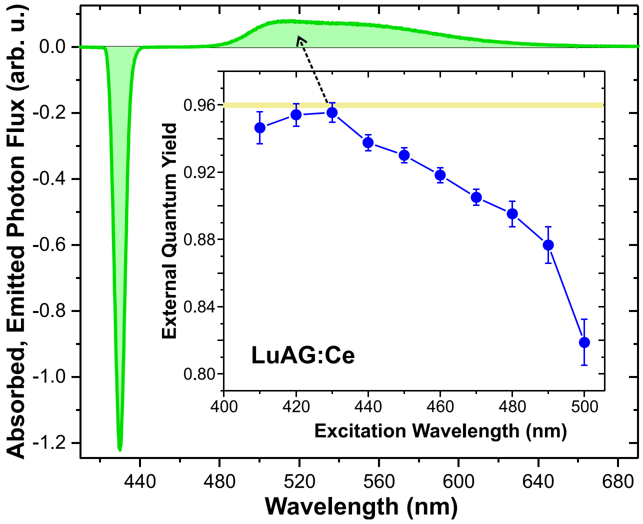

Figure S7. PLQY excitation dependence for the standard sample of LuAG:Ce, which serves for cross-checking of the setup performance. The absorbed/emitted photon flux for the 430 nm excitation is determined from the green curve as the positive peak area (emitted photon flux) divided by the negative peak area (absorbed photon flux).

5. Effects of photon recycling

In order to demonstrate the importance of photon recycling (PR), we performed new experiments. PL spectra of the stock concentration of DDAB(2) sample were compared with the diluted sample (3-times lower volume concentration). PL was excited by the fiber-coupled LED at 390 nm. The measurements were performed either without or with the integrating sphere. Figure S8 demonstrates that the photon recycling effect is strongly amplified inside the integrating sphere. The shift due to PR is up to 30 meV.

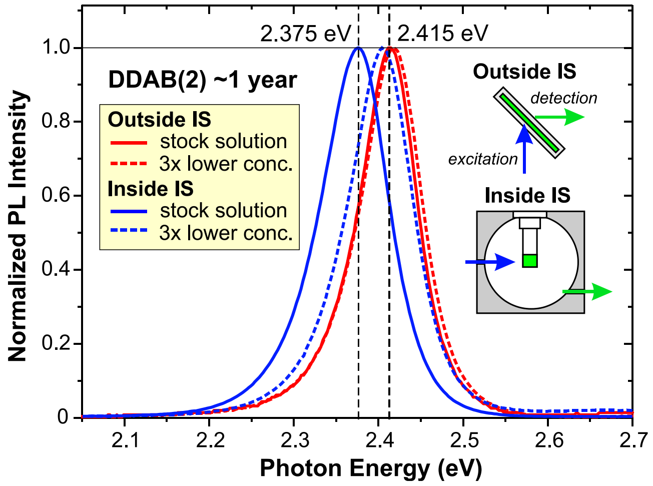

Figure S8. Normalized PL spectra under excitation at 390 nm. The stock concentration and 3x diluted samples are measured either outside IS in 1-mm cuvettes (red lines) or inside IS in the standard vial flasks (blue lines) – the configurations are schematically represented on the right side of the figure.

6. Comparing PLQY of OA and DDAB passivated and optimally aged samples

The difference between OA and DDAB passivated samples is clearly apparent during the aging (Fig. 3). The OA-passivated samples are always inferior to the DDAB-passivated samples. This fact is illustrated by PLQY curves obtained for the optimally aged samples (around 90 days) in Fig. S9. Both SPL and ASPL (and consequently the total PLQY) yields are smaller for OA-passivated sample. While the SPL peak yield difference is just around 11%, for ASPL it is about 22%.

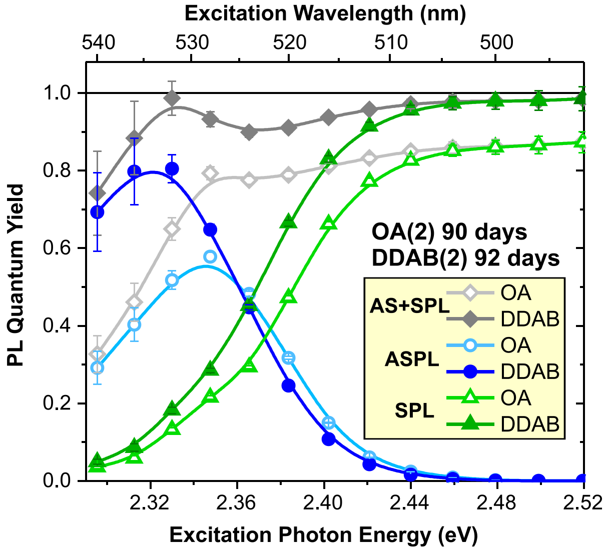

Figure S9. Comparison of PLQY for samples passivated by OA and DDAB after an optimal aging of about 90 days.

References

1. Protesescu, L.; Yakunin, S.; Bodnarchuk, M. I.; Krieg, F.; Caputo, R.; Hendon, C. H.; Yang, R. X.; Walsh, A.; Kovalenko, M. V. Nanocrystals of Cesium Lead Halide Perovskites ($CsPbX_3$, X = Cl, Br, and I): Novel Optoelectronic Materials Showing Bright Emission with Wide Color Gamut. *Nano Lett.* **2015**, 15, 3692–3696.

2. M. I. Bodnarchuk, S. C. Boehme, S. ten Brinck, C. Bernasconi, Y. Shynkarenko, F. Krieg, R. Widmer, B. Aeschlimann, D. Günther, M. V. Kovalenko and I. Infante. Rationalizing and controlling the surface structure and electronic passivation of cesium lead halide nanocrystals. *ACS Energy Lett*. **2019**, 4, 63–74.

3. Maes, J.; Balcaen, L.; Drijvers, E.; Zhao, Q.; De Roo, J.; Vantomme, A.; Vanhaecke, F.; Geiregat, P.; Hens, Z. Light Absorption Coefficient of $CsPbBr_3$ Perovskite Nanocrystals. *J. Phys. Chem. Lett.* **2018**, 9, 3093–3097.

4. Greben, M.; Khoroshyy, P.; Sychugov, I.; Valenta J. Non-exponential decay kinetics: Correct assessment and description illustrated by slow luminescence of Si nanostructure. Appl. Spectr. Reviews **2019**, 54, 758–801.

5. CRYTUR: Cryphosphor^{TM} Datasheet.
